# A systematic review on the impact of nutrition and possible supplementation on the deficiency of vitamin complexes, iron, omega-3-fatty acids, and lycopene in relation to increased morbidity in women after menopause

**DOI:** 10.1007/s00404-024-07555-6

**Published:** 2024-06-27

**Authors:** Friederike Wylenzek, Kai J. Bühling, Elena Laakmann

**Affiliations:** 1 Department of Gynecological Endocrinology, University Medical Center Hamburg-Eppendorf, Martinistrasse 52, 20246 Hamburg, Germany; 2 Department of Gynecology, University Medical Center Hamburg-Eppendorf, Martinistrasse 52, 20246 Hamburg, Germany

**Keywords:** Postmenopause, Vitamin deficiency, Chronic diseases, B-vitamins, Omega-3-fatty acids, Vitamin D, Lycopene, Iron, Micronutrients, Diet, Osteoporosis, Neurodegenerative and cardiovascular diseases

## Abstract

**Summary:**

A balanced and healthy diet during the menopausal transition and after menopause is crucial for women to reduce the risk for morbidities and chronic diseases due to deficiency of essential nutrients.

**Purpose:**

The objective of this study was to conduct a systematic review of studies that analyzed the impact of vitamin and nutrient deficiencies in postmenopausal women in relation to increased morbidities and chronic conditions.

**Methods:**

Observational studies were searched in the databases PubMed, UpToDate, and Google Scholar.

**Results:**

We searched 122 studies, of which 90 were included in our analysis. The meta-analysis of the data could not be performed because of the heterogeneity of the statistical methods in the included studies. In our study, we focused on the aspects of vitamin B6, vitamin B12, vitamin D, iron, omega-3-fatty acids, and lycopene, belonging to the family of carotenoids. Postmenopausal women with deficiencies of these nutrients are more vulnerable to comorbidities such as cardiovascular and cerebrovascular events, metabolic diseases, osteoporosis, obesity, cancer and neurodegenerative diseases such as Parkinson’s disease, Alzheimer’s disease, depression, cognitive decline, dementia, and stroke. We concluded that women after menopause tend to have a greater probability of suffering from deficiencies in various vitamins and nutrients, and consequently have an increased risk of developing morbidities and chronic diseases.

**Conclusion:**

In conclusion, maintaining optimum serum levels of nutrients and vitamins, either through a balanced and healthy diet consuming fresh fruits, vegetables, and fats or by taking appropriate supplementation, is essential in maintaining optimal health-related quality of life and reducing the risk for women during the menopausal transition and after menopause. Nevertheless, more recent studies need to be assessed to formulate adequate recommendations to achieve positive clinical outcomes.

## What does this study add to the clinical work

**Table:** 

Women during the menopausal transition and after menopause are at an increased risk of having deficiencies or changing levels of vitamins and nutrients. This article provides and overview of the possible consequences and recommendations to reduce risk of developing morbidities and chronic conditions due to unbalanced levels of nutrients.	

## Introduction

Menopause is divided into three stages: (1) perimenopause or menopausal transition which is the time interval of decreasing ovarian function resulting in irregular menstruation cycles, (2) menopause, which is the permanent cessation of menstruation which is diagnosed after 12 months of amenorrhea and can only be retrospectively detected, and (3) postmenopause, which begins 12 months after last menstruation [1].

Vitamins play a crucial role in human homeostasis; therefore, adequate levels of vitamins, ferritin, lycopene, and omega-3-fatty acids are important to preserve an optimal health-related quality of life [2].

**Fig. 1:**
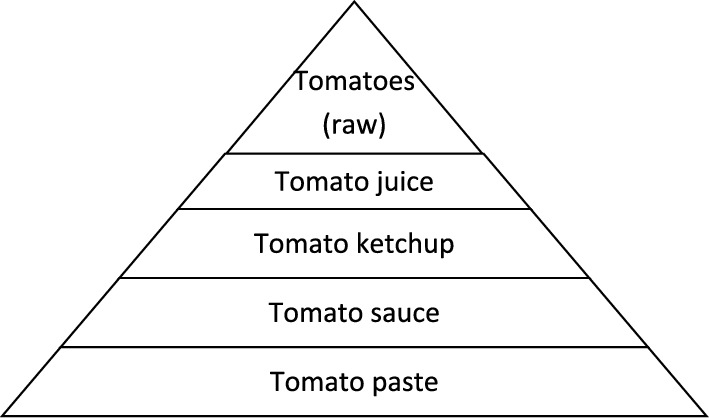
Distribution of Lycopene content in different tomato products [10]

While with increasing age in men and women, energy needs decrease, micronutrient requirements stay the same, and thus, menopausal women require specific food that provides a higher nutrient density to obtain an optimal level of vitamins and micronutrients. Therefore, through menopausal transition, levels of these nutrients are likely to change, thus bringing an imbalance into the woman's body and as a consequence, leading to an increased risk for morbidities. Consequently, women are more vulnerable to comorbidities such as cardiovascular events, metabolic diseases, osteoporosis, obesity, cognitive decline, depression, dementia, and cancer [3, 4] (Fig. 1).

**Fig. 2:**
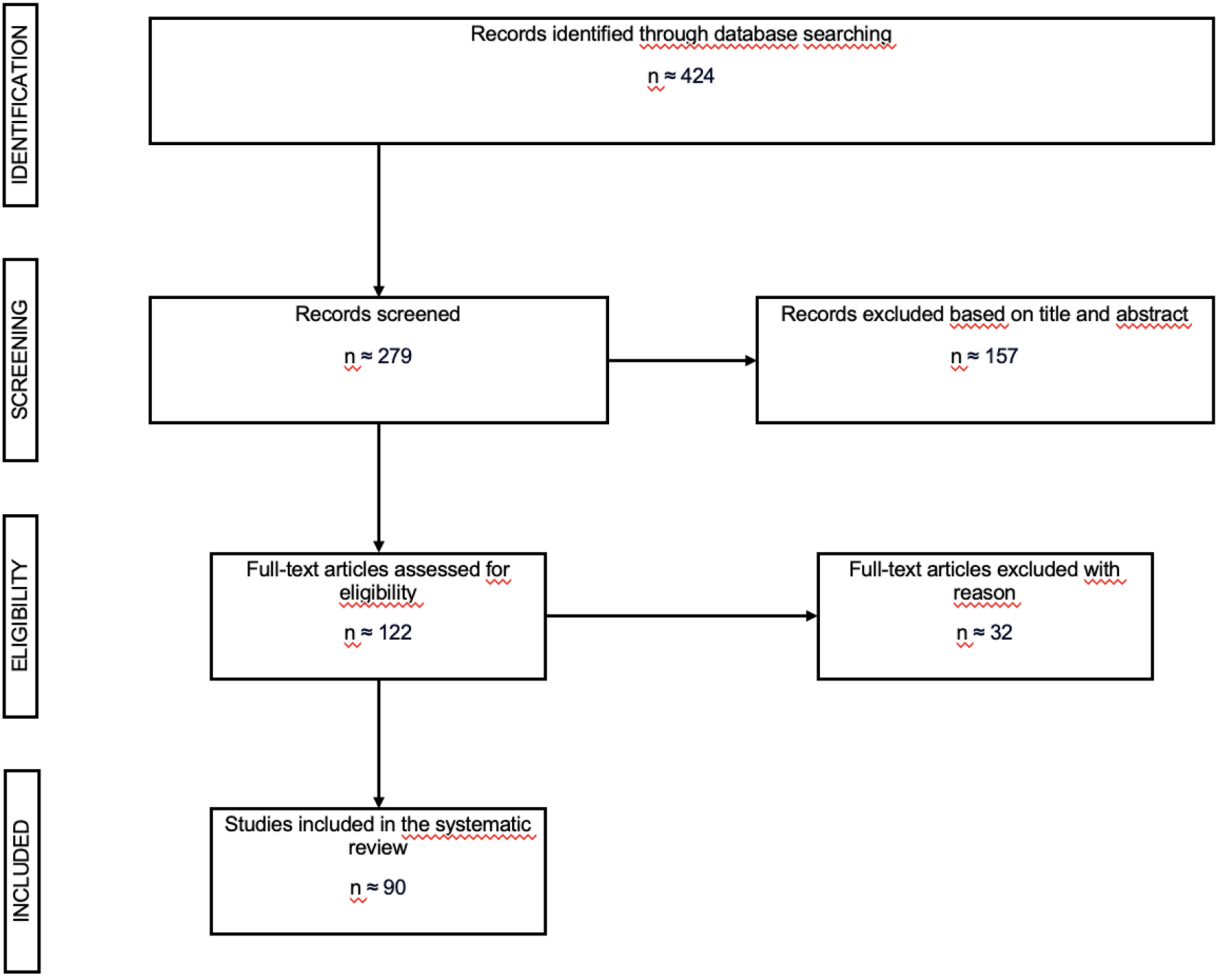
PRISMA flow chart of literature search

As dietary supplements are over-the-counter medicine and no prescription is needed, supplements such as vitamins, minerals, carotenoids, and omega-3-fatty acids are widely used in Western countries. 25 percent of German women take supplements daily for the prevention of chronic diseases and the proportion of postmenopausal women taking supplements regularly is even higher [4] (Fig. 2).

Vitamin complexes, iron, lycopene, and omega-3-fatty acids are essential nutrients that perform important functions in different body systems and are therefore crucial for maintaining optimal health [3, 5, 6] (Table 1).

**Table 1:** Selected types of fruits and vegetables with the amount of Lycopene in µg/g wet weight [10]

Fruits and vegetables	Lycopene (µg/g wet weight)
Tomatoes	8,8–42,0
Watermelon	23,0–72,0
Pink guava	54,0
Pink grapefruit	33,6
Papaya	20,0–53,0
Apricot	< 0,1

Vitamin B complexes are crucial in energy production, mood and mental stability, cardiovascular and cerebrovascular, as well as neurodegenerative diseases. The fat-soluble vitamin D is a vitamin that the body can either synthesize on its own after sun exposure or is ingested with food or supplements. Vitamin D is responsible for many essential functions in the human body, including the maintenance of serum levels of calcium and phosphorus concentration, regulating intestinal calcium absorption, as well as bone and renal calcium resorption, and is also important for skeletal mineralization and the regulation of parathyroid hormone production [1, 5] (Table 2).

**Table 2:** Daily Recommendation of nutrients in postmenopausal women [16–19]

Nutrient	Daily recommendation in postmenopausal women
Vitamin B6	1,5 mg/d
Vitamin B12	2,4 µg/d
Vitamin D	20 µg/d (800 IU)
Iron	8 mg/d
Omega-3-fatty acids	2–4 g/d
Lycopene	> 30 mg/d

Iron is an essential trace element that the body requires for proper function and development and is involved in a wide range of physiological and pathological processes. Ferritin serves as the most widely used serum marker of total body iron stores [7, 8]. Iron is second to estrogen in terms of importance for a woman’s body development. Iron, an important growth nutrient, is needed for oxygen transport, DNA synthesis, and energy production [9].

Fatty acids are crucial for many systems in the body, such as the cardiovascular, pulmonary, immune and endocrine systems, as well as being involved in the brain and eyes. Furthermore, omega-3-fatty acids have anti-inflammatory, cardio-protective and insulin-sensitizing effects, as well as bone-protective properties [1].

Carotenoids are biopigments that are synthesized by plants and microorganisms. Fruits and vegetables are the main sources of carotenoids in the human diet [10, 11]. Carotenoids have many different beneficial and protective functions and therefore are helpful in preventing chronic diseases, including cardiovascular diseases, certain cancer types, and other chronic conditions [10, 12, 13].

The phytochemical lycopene plays a very important role in the carotenoid family. Lycopene is a lipid-soluble antioxidant that is responsible for the red color of different vegetables and fruits, especially tomatoes. Lycopene is absorbed in the human body across the gastrointestinal tract via a chylomicron mechanism [10, 14]. Processed tomato products absorb lycopene more efficiently compared with raw tomato products [10, 15]Tomatoes, watermelon, pink guava, pink grapefruit, papaya, and apricot are vegetables and fruits that are very rich in lycopene [10].

## Materials and methods

### Search strategy

This systematic review is based on database research of cross-sectional studies, randomized-controlled studies, meta-analyses, and systematic reviews on micronutrient deficiencies and their correlation with risk of chronic diseases in postmenopausal women. The data search was performed via PubMed, UpToDate, and Google Scholar databases. The systematic review was performed according to the PRISMA 2020 Statement (Table 3).

**Table 3:** The applied criteria for a population, intervention/exposure, comparator, outcome and study design (PICOS)

PICOS	Inclusion criteria	Exclusion criteria
Population	Women in menopausal transition/ postmenopausal women	Women before menopause/ mixed populations
Intervention/Exposure	Vitamin and micronutrient deficiency after menopause	Vitamin and micronutrient deficiency concerning not the appropriate population
Comparison	Effectiveness of dietary intervention/ supplementation in relation to increased morbidity after menopause compared to non-adapted dietary habits	Effectiveness of dietary intervention/ supplementation influenced by interfering variables
Outcome	Decreased risk of developing morbidities due to insufficient vitamin and micronutrient levels related to menopause	Outcomes concerning mixed populations
Study design	Cross-sectional studies, randomized controlled studies, meta-analyses, and systematic reviews	Studies not published in either English or German languages

### Inclusion criteria

Studies carried out in women during the menopausal transition;

Studies carried out in women after menopause;

Studies published either in English or German languages;

Studies carried out with ethical approval;

Dietary intervention applied within the studies while using either appropriate food products or supplementation.

### Exclusion criteria

Studies carried out in a mixed population (female/male);

Studies carried out in a group of premenopausal women;

Studies carried out in animal models;

Studies with inaccurate descriptions of methodology and results.

### Selection process

After the above-described search strategy, a total of 122 publications with relevant titles were found. From the search results, the studies that met the mentioned criteria were selected for inclusion in the study.

## Results

We identified 122 studies. Ninety met the inclusion criteria and were used for our study.

### Vitamin B6

The study by Dennehy and Tsourounis shows that high levels of homocysteine caused by vitamin B6 deficiency can be seen in postmenopausal women [5]. In 2006, Ramakrishnan et al*.* revealed elevated homocysteine levels in vitamin B6 deficiencies [20]. Elevated homocysteine levels found by Boushey increase the risk of cardiovascular and cerebrovascular diseases [21]. These results are confirmed by the study of Shenoy et al*.* for the severity of coronary diseases [22]. Fasting serum homocysteine levels were significantly elevated in patients with coronary artery disease compared to patients without coronary artery disease (*p* < 0.001) [23]; additionally, Shenoy et al*.* stated that the level of elevated homocysteine is correlated with the severity of coronary artery disease (*p* < 0,001) [22].

As vitamin B6 is involved in the synthesis of certain neurotransmitters, such as serotonin and dopamine, it plays a crucial role in the regulation of mood and mental function. Therefore, studies by Hvas et al*.* and Dennehy and Tsourounis, as well as a meta-analysis and systematic review by Wu et al*.* suggest that a decreased level of vitamin B6 might contribute to the development of depressive symptoms [5, 24, 25]. Supplementation of vitamin B6 decreases the level of circulating homocysteine [5, 26, 27].

Dietary sources of vitamin B6 include whole unprocessed foods including beans, meat, fish, and certain fruits and vegetables as well as fortified cereals [5].

### Vitamin B12

One of the major risk factors for coronary artery disease is endothelial dysfunctional atherosclerosis. As vitamin B12 and folic acid are involved in the metabolic processes of homocysteine, hyperhomocysteinemia may lead to a deficiency in folic acid and vitamin B12 and result in an increased risk for cardiovascular diseases, stroke, and Alzheimer’s [5, 28, 29].

These statements are reinforced by Selhub et al*.*, Dennehy and Tsourounis, and Butola et al*.* [5, 29, 30].

Selhub explains that a deficiency of vitamin B12 over a constant period might consequently lead to cognitive dysfunction [30]. These investigations are reinforced by Boushey, Seshardri et al*.* and Butola et al*.* who suggest that in postmenopausal women, low levels of vitamin B12 and high levels of homocysteine cause oxidative stress which over time lead to silent injuries in the brain, thus resulting in calcium inflow and apoptosis [21, 29, 31]. Therefore, prolonged low levels of vitamin B12 can lead to serious cognitive dysfunction [30].

Butola et al*.* provided evidence for the associations between vitamin B12 deficiency and decreased bone mineral density, leading to increased elderly fragility and disability [29]. They highlighted the relationship between low levels of vitamin B2, B12, folic acid, and homocysteine.

High levels of homocysteine affect bone blood flow, increase the metalloproteinase matrix, and interfere with the cross-linking of collagen, which is essential as collagen cross-linking provides stability and strength to the bone matrix collagen network [29, 32].

Reynolds, Penninx as well as Hanna, Lachover and Rajarethinam [33–35], and Butola et al*.* [29] showed associations between vitamin B12 deficiency and anemia, neuropathy and neuropsychiatric diseases. Butola et al*.* mentioned the association between low B12 levels and neurological symptoms such as tingling sensations in the hands and feet and possible peripheral nerve damage leading to problems in movement and coordination [29].

As dementia, depression and mental impairment are associated with a deficiency in vitamin B12, Hanna, Lachover, and Rajarethinam mentioned the involvement of vitamin B12 and folic acid in the synthesis of monoamines, such as dopamine and serotonin, that are implicated in the pathophysiology of neuropsychiatric disorders such as depression and psychosis [35]. Furthermore, Butola et al*.* stated that age-related macular degeneration results from vitamin B12 deficiency [29]. Zampatt et al*.* in the Blue Mountain Eye study, compared the levels between homocysteine, vitamin B12, and folic acid and concluded that serum homocysteine levels > 15 µmol/l are associated with a greater risk for age-related macular degeneration, which accounts for one of the major causes for disability due to loss of vision. They mentioned a randomized trial of 5442 females in which participants who were supplemented with vitamin B12, B9, B6, and folic acid daily showed a decreased risk of age-related macular degeneration [36].

### Vitamin D

In 2020, Pérez-López, Chedraui, and Pilz identified that postmenopausal women are at an increased risk for vitamin D deficiency [37]. Ko and Kim found an association between vitamin-D-deficient postmenopausal women and an alteration in lipid profile [1]. This was also shown by Pérez-López, Chedraui, and Pilz, who provided evidence suggesting that low levels of vitamin D are linked to metabolic syndrome, high triglyceride levels, and low HDL cholesterol levels. Pérez-López, Chedraui, and Pilz as well as Ko and Kim highlighted the inverse relationship between serum levels of 25(OH)D and fat mass, and in 2007, Holick proved that obesity diminishes the bioavailability of serum vitamin D levels [1, 37, 38]. Ko and Kim referred to a cross-sectional study suggesting that obese postmenopausal women are more vulnerable to vitamin D deficiency because high serum 25(OH)D levels inversely correlate with BMI, adiposity and waist–hip ratio [1]. Pérez-López, Chedraui, and Pilz reinforced this statement by referring to a double-blind RCT suggesting that daily supplementation of 1000 IU of vitamin D in postmenopausal women positively affects metabolic risk profiles and increases serum 25(OH)D levels. Vitamin D deficiency increases the risk for the development of secondary hyperparathyroidism, high bone turnover, bone loss, mineralization defects, muscle weakness, and postmenopausal osteoporosis resulting in higher fracture risk [2].

Menopause is the biggest risk factor for osteoporosis [39] and more than 50% of postmenopausal women take medication due to suboptimal levels of 25(OH)D to prevent osteoporosis [38]. Dennehy and Tsourounis suggested in their study that daily supplementation of 700–800 IU of vitamin D3 and 500–1200 mg of calcium may significantly increase the BMD of the total body, spine, femoral neck, and hip, promote stability and muscle strength, and prevent postmenopausal osteoporosis, thus reducing fracture risk in postmenopausal women [5].

Rizzoli et al*.* referred to recommendations posed by the European guidance for the diagnosis and management of osteoporosis in postmenopausal women, i.e., daily intake of 800 IU of vitamin D, 1000 mg/day of calcium, and 1 g/kg body weight of protein for all women aged over 50 years [39]. With increasing age, vitamin D3 concentration is much lower and less efficient in elderly women compared with young adults. This is due to insufficient sunlight exposure and a decrease in the functional capacity of the skin to synthesize vitamin D3 under the influence of UV light [40].

The studies by Lee et al*.,* Anderson et al., Dennehy and Tsourounis, as well as Mozos and Marginean showed that low vitamin D levels in postmenopausal women are linked to increased risk of cardiovascular events [5, 41–43].

### Iron

In 2000, Zacharski et al*.* (according to NHANES III) detected an inverse relationship between decreasing estrogen levels and increasing iron levels during the menopausal transition [44]. As estrogen decreases due to the cessation of ovarian functions, ferritin levels increase in concentration when comparing pre- and postmenopausal women as the result of decreasing menstrual periods and, therefore, elevated ferritin levels are very likely to affect the health of postmenopausal women. The massive decline in estrogen during the menopausal transition is the main causative factor for climacteric symptoms [44–46]. Jian, Pelle, and Huang in the NHANES III study demonstrated that women who experience hot flashes during the menopausal transition also have elevated serum ferritin levels [46]. In 2009 Jian, Pelle and Huang, in 2015 Ma et al*.,* and in 2018 Zhang et al*.* described a two-to-three-fold increase in serum ferritin levels during the menopausal transition [8, 46, 47].

Ma et al*.* showed an association between high serum ferritin levels and increased cardiovascular events in postmenopausal women [8]. This was likewise confirmed by Zacharski, Shamayeva, and Chow [48]. Excess iron most often accumulates in the heart and the liver, leading to injury due to chronic free radicals and eventually causing progressive heart and liver failure [7]. Elevated iron levels in postmenopausal women contribute to an increased risk of cardiovascular events, breast cancer via oxidative-stress pathways and osteoporosis [49–51]. This was shown by Jian et al*.* who provided evidence suggesting that high levels of iron and estrogen are crucial in the incidence of low-grade estrogen-receptor-positive breast cancer [52].

While iron increases during menopause, the skin is exposed to a greater amount of iron therefore leading to oxidative stress that consequently makes the skin more vulnerable to UV damage and expedites the aging process [46].

Yamasaki and Hagiwara demonstrated that osteoporosis can be caused by high iron levels due to its inhibitory effect on osteoblastic proliferation and differentiation [53]. In 2012, Li et al*.* presented the same outcome—iron overload being linked to increased bone resorption, oxidative stress, and altered bone microarchitecture, consequently causing bone density loss [51]. The study by Chen et al*.* provided evidence suggesting that postmenopausal women with fractures have decreased bone mineral density and increased serum ferritin levels [54]. Chen et al*.* referred to their retrospective study in which they analyzed women aged older than 70 years with a hip fracture who showed elevated serum ferritin levels together with significantly reduced bone mineral density. They pointed to a team of scientists in Seoul that analyzed a 3-year longitudinal health promotion center-based study, in which they assessed 789 middle-aged men and 940 postmenopausal women. They found a linear association between increased serum ferritin levels and vertebral fracture prevalence in postmenopausal women, whereas these correlations could not be detected in the male control group. They also highlighted other studies that demonstrated that in healthy women, aged older than 47 years, an association between elevated serum ferritin levels and an accelerated rate of bone loss was found, therefore suggesting that women are at an increased risk of osteoporosis during their menopausal transition and that their serum ferritin levels should be regularly checked [54].

Several studies show the crucial role of hepcidin, a regulator of iron homeostasis, in the development of postmenopausal osteoporosis. Li et al*.* explained that through the binding of hepcidin to the iron transport protein, ferroportin, and the consequent reduction in ferroportin activity, iron uptake and transport from the gastrointestinal system to the circulatory system could be reduced and inhibited [51]. He referred to the study by Rivera et al*.* that showed a 70% reduction of serum iron levels that lasted for 3 days in mice that received injections of human hepcidin [51, 55]. Li et al*.* demonstrated that hepcidin could play a promising role in the prevention and treatment of osteoporosis and referred to deferiprone, as hepcidin is not a drug [51]. Zhang et al*.* also provided evidence for the beneficial effects of hepcidin in the management of osteoporosis [47].

### Omega-3-fatty acids

In 2014 Koren et al*.* and Shen et al*.* provided evidence supporting the effect of omega-3 fatty acids on bone health due to their anti-inflammatory characteristics [56, 57]. Omega-3 fatty acids are involved in the suppression of bone resorption and prevention of bone loss [57, 58]. As postmenopausal women are susceptible to osteoporosis, which is characterized by decreased bone mineral density, increased bone turnover, and therefore a higher risk of fractures, omega-3 fatty acids may have a slight decreasing effect on bone turnover markers [57]. Kruger and Horrobin showed a positive effect of fatty acids in the absorption of calcium and the prevention of urinary calcium loss [59]. This was also evidenced in 2007 by Griel et al. and Shen et al. [57, 60].

Studies by Fenton et al*.,* Shen et al., and Griel et al. all demonstrated beneficial health effects of omega-3 fatty acids on cognitive performance, prevention of cardiovascular and cerebrovascular diseases, and support of the immune system [57, 60, 61]. A study in 2013 by Luchtman and Song likewise evidenced that sufficient intake of omega-3 fatty acids restores cognitive function [62].

### Lycopene

Studies have established the positive link between a balanced diet and higher tissue concentration of carotenoids, consequently lowering the risk of developing chronic diseases and certain types of cancer [14, 63, 64].

The major functions of the potent antioxidant lycopene are to remove free radicals, alleviate oxidative stress, modulate immune function, prevent cardiovascular diseases, improve bone health, and prevent postmenopausal osteoporosis and hypertension [65–70]. Several reports show that lycopene can prevent cardiovascular diseases [10, 69]. Epidemiological studies highlight the inverse association between low levels of lycopene and the incidence of coronary heart diseases. Fuhrman, Elis, Aviram as well as Rao, Ray, L.G. Rao and Rao, Rao reported that lycopene can reduce oxidized LDL and serum total cholesterol levels thus lowering the risk of cardiovascular diseases [10, 65, 69]. Furthermore, associations between hypertension and oxidative stress are recognized; thus, several studies have reported that lycopene significantly reduces elevated blood pressure [10, 67, 68].

Lycopene has anti-tumor effects and decreases the risk of certain cancer types through its protection of cells from oxidative stress and damage [69–71]. Additionally, Palozza et al*.* in 2011 and Peng et al*.* in 2017 provided evidence for lycopene’s protective features by regulating growth factor signaling, cell cycle arrest, induction of cell cycle apoptosis, and inhibition of cell invasion, angiogenesis, and metastasis [71, 72]. Some types of cancer in which lycopene might have a protective role are breast, cervical, ovarian, liver, lung, and prostate [10]. Levi e*t al.*, Hultén e*t al.,* and Sato et al*.* provided evidence for strong inverse associations between serum lycopene levels and breast cancer risk [72–74]. Peng et al*.* concluded that lycopene has a positive effect on MCF-7 human breast cancer cells. They treated MCF-7 cells with lycopene at different concentrations and for different durations. The number of MCF-7 cells showed a significant decrease as well as shrinkage and breakage after lycopene treatment with significant results while increasing concentration and prolonging treatment duration. They concluded that lycopene arrests cells at the G0/G1 phase, causing inhibition of MCF-7 cell proliferation and inducing cell apoptosis, thus giving evidence that lycopene has anti-tumor effects on estrogen receptor-positive breast cancer cells [75]. In 2015 and 2017, respectively, Asbaghi et al*.* and Peng et al. stated that patients with pulmonary cancer showed significantly lower serum lycopene levels compared with healthy individuals [75, 76].

Studies report that lycopene has a positive effect on bone health due to its stimulatory effect on cell proliferation, on differentiation marker alkaline phosphatase of osteoblasts, and on its inhibition effect on osteoclast formation as well as resorption [77–79]. Rao et al*.* presented a direct correlation between serum lycopene levels in postmenopausal women and oxidative stress and bone turnover markers; therefore, antioxidants may counteract oxidative stress that is associated with the development of osteoporosis [77–79]. Walallawita et al*.* suggested that lycopene shows positive properties in preventing bone loss in postmenopausal women and referred to the beneficial effect of proper lycopene intake and the reduction of bone resorption markers [19].

Oxidative stress is a causative agent that plays a crucial role in the pathogenesis of neurodegenerative diseases, such as Parkinson’s disease, Alzheimer’s disease, depression, and stroke. Different studies report that significantly low levels of lycopene are seen in patients with Parkinson’s disease and vascular dementia [80]. Low levels of lycopene increase the risk of cerebral microangiopathy [81] and indicate the possible protective role of lycopene concerning the development of amyotrophic lateral sclerosis [82]. The studies by Chen et al*.* and Saini et al*.* provided evidence suggesting that inhibiting oxidative stress, neuroinflammation, and suppressing neuronal apoptosis have therapeutic and prophylactic effects on various neurogenerative diseases and can restore mitochondrial function [83, 84]. As an antioxidant, lycopene is essential in the prevention of many diseases and contributes to an improvement in quality of life [70].

## Discussion

Women during the menopausal transition and after menopause are at an increased risk of having deficiencies or changing levels of vitamins and nutrients. Due to unbalanced levels of these nutrients, postmenopausal women are more vulnerable to developing morbidities and chronic conditions compared with premenopausal women.

### B vitamins

Studies by Dennehy and Tsourounis as well as Ramakrishnan et al*.* suggested that in postmenopausal women, vitamin B6 deficiency can lead to elevated levels of the amino acid homocysteine [5, 20].

Selhub et al*.* referred to a case–control study of 164 patients confirmed with dementia of the Alzheimer’s type in which patients showed a significant increase in homocysteine and low levels of vitamin B12 and folic acid [30].

Osteoporosis affects one-third of postmenopausal women; therefore, menopause is the biggest risk factor for osteoporosis in women aged over 50 years [39]. Butola et al*.* analyzed an association between low levels of vitamin B12 and decreased bone mineral density and a consequently increased risk of fragility and elderly disability [29].

### Vitamin D

Postmenopausal women are at an increased risk of developing vitamin D deficiency, which is linked to diet, lifestyle, changes in body composition, insulin sensitivity, and reduced physical activity [37].

Pérez-López, Chedraui, and Pilz referred to a double-blind RCT from a Brazilian research group, which showed that daily supplementation of 1000 IU of vitamin D3 in 50–65 year old postmenopausal women in a time interval of 9 months positively affected metabolic risk profile and a significant increase (+ 45.4%) of serum 25(OH)D levels in women that had a regular intake of vitamin D supplement compared with the placebo group, which showed a decrease (− 18.5%) in serum levels of vitamin D (*p* = 0.0049) [37]. Ko and Kim reported a cross-sectional analysis of 292 postmenopausal women, which suggested that higher serum 25(OH)D levels are inversely correlated with BMI, adiposity, and waist–hip ratio; thus, obese postmenopausal women are more vulnerable to low serum vitamin D levels [1]. Pérez-López, Chedraui, and Pilz reported that postmenopausal women are highly vulnerable to develop a Vitamin D deficiency [37].

Regarding a relationship between vitamin D deficiency and breast cancer or cognitive decline, the results are either very controversial or show no association [1, 85, 86]. Wiacek et al*.* concluded in their study that an age-dependent rise in serum levels of vitamin D is not significant enough to prevent age-dependent diseases including postmenopausal osteoporosis and/or Alzheimer’s disease [2, 86].

### Iron

High levels of serum ferritin are associated with several health problems. Therefore, postmenopausal women are at an increased risk of cardiovascular events. The study by Ma et al*.* investigated a total number of 1178 Chinese postmenopausal women and they concluded elevated serum ferritin levels have a relatively strong positive correlation with cardiovascular events, such as carotid atherosclerosis. They highlighted that the deposition of ferritin in the blood vessels can be involved with the oxidation of lipids and formation of oxidized LDL-cholesterol, thus contributing to the development of foam cells and the progression of atherosclerosis [8]. Nevertheless, the theory of association between elevated serum ferritin levels and the risk of developing cardiovascular diseases remains controversial. Ma et al*.* referred to some studies that point out contrasting statements. A randomized, controlled, single-blinded clinical trial with 1277 participants proved no statistical significance in the reduction in mortality, nonfatal myocardial infarction, or stroke when reducing ferritin levels in patients with symptomatic peripheral artery disease [8, 87]. In contrast to the theory by Zacharski et al., Zacharski, Shamayeva, and Chow provided evidence that a reduction in serum ferritin levels can reduce cardiovascular risk and a low ferritin burden predicts improved outcomes [8, 48]. Ma et al*.* suggested that postmenopausal women with increased serum ferritin levels should receive management of cardiovascular risk factors to prevent cardiac-related health problems [8].

Besides the potential increased cardiovascular risk in relation to elevated serum ferritin levels, Huang suggested that increased concentrations of iron contribute to increased vulnerability to breast cancer via the oxidative-stress pathway in postmenopausal women [50]. This statement is strengthened by a study from Jian et al*.*, in which enhanced cell proliferation in estrogen-receptor-positive cells was shown due to high levels of estradiol and ferritin; therefore, he concluded that high levels of iron in postmenopausal women together with estrogen levels might play a crucial role in the incidence of low-grade but estrogen-receptor-positive breast cancer [52].

### Omega-3-fatty acids

As omega-3 fatty acids have many different beneficial health effects, the positive effect on bone health shows conflicting evidence [57]. Omega-3 fatty acids have beneficial effects on bone health due to their anti-inflammatory actions [56]. Nevertheless, statements concerning the positive effect on bone turnover markers are very controversial. Several RCTs which were included in the meta-analysis by Shen et al*.* provide evidence that the intervention of omega-3 fatty acids significantly reduces bone formation marker osteocalcin, but the efficacy in affecting serum bone-specific alkaline phosphatase, a bone formation marker, and collagen type-I cross-linked C-telopeptide, a bone resorption marker, did not show statistical significance [57]. Controversially, Kajarabille et al*.* demonstrated in the systematic review evidence for the supporting benefits of omega-3 fatty acids on bone turnover markers and bone health [58]. Kajarabille et al*.* and Shen et al*.* highlighted that omega-3 fatty acids are involved in the suppression of bone resorption and prevention of serious bone loss, because omega-3-fatty acids decrease prostaglandin E2, which in turn decreases nuclear factor-κ B ligand (RANKL) expression and increases osteoprotegerin production [57, 58]. Besides the effects on bone health, omega-3 fatty acids play a crucial role and have numerous beneficial health effects on cognitive performance and the prevention of cardiovascular and cerebrovascular diseases and are also involved in the immune system [57, 61]. A sufficient supply of omega-3 fatty acids may help to restore cognitive function [62]. Therefore, it is important to consume proper levels of omega-3 fatty acids regularly due to their numerous beneficial health effects [57].

### Lycopene

Several studies provide evidence for the positive correlation between higher dietary intake as well as tissue concentration of carotenoids and lower risk of chronic diseases [10, 14, 63]. As lycopene is a potent antioxidant, it can protect cells from oxidative stress and damage, thus reducing the risk of several types of cancer. Positive associations between increased serum levels of lycopene and significantly reduced risk for cancer have been demonstrated by different studies [14, 69, 71]. The effect of lycopene in reducing the risk of breast cancer remains controversial. Some studies show no positive effect [88–90], while other studies establish a positive correlation between lycopene intake and reduced risk of developing breast cancer [90]. A Swiss case–control study with 289 cases by Levi et al [72], a case–control study of 295 U.S. women by Sato et al*.* [74], and a nested case–control study of Swedish postmenopausal women [73] all reported a strong inverse association between serum lycopene levels and breast cancer risk.

## Conclusion

In conclusion, maintaining optimum serum levels of nutrients and vitamins, either through a balanced and healthy diet consuming fresh fruits, vegetables, and fats or by taking appropriate supplementation, is essential in maintaining optimal health-related quality of life and reducing the risk for women during the menopausal transition and after menopause. Confirmed evidence of the benefit of the supplementation of vitamin B6, vitamin B12, and vitamin D exists regarding the improvement of cardiovascular and cerebrovascular diseases, neurological symptoms, metabolic risk profiles, and osteoporosis. Appropriate levels of iron positively improve climacteric symptoms, decrease the risk of cardiovascular diseases, including heart failure and liver failure, and decrease risk of breast cancer and osteoporosis. Supplementing omega-3-fatty acids benefits the immune system and improves cognitive performance and outcomes for osteoporosis and cardiovascular/cerebrovascular disease. Lycopene removes free radicals, alleviates oxidative stress, benefits bone health, prevents postmenopausal osteoporosis and hypertension, and decreases the risk of certain cancer types.

However, the importance and effect of optimal dietary habits and nutritional supplements in menopausal women or women in menopausal transition needs to be explored further and should be addressed in future studies.

Maintaining an optimal level of daily nutritional intake should be considered as an important topic for further research, as decreasing the risk of developing chronic diseases plays, by implication, a significant role in the economic health system.

## Data Availability

The data that support the findings of this study are openly available in Google Scholar, PubMed and UpToDate. The associated DOI number is linked to each reference.

## References

[1] Ko SH, Kim HS (2020) Menopause-associated lipid metabolic disorders and foods beneficial for postmenopausal women. Nutrients 12(1):202 31941004 10.3390/nu12010202 PMC7019719

[2] Wiacek M, Zubrzycki IZ, Bojke O, Kim HJ (2013) Menopause and age-driven changes in blood level of fat- and water-soluble vitamins. Climacteric Dezember 16(6):689–699 10.3109/13697137.2012.742504 23215463

[3] Lobo RA, Davis SR, De Villiers TJ, Gompel A, Henderson VW, Hodis HN (2014) U. a. prevention of diseases after menopause. Climacteric 17(5):540–556 24969415 10.3109/13697137.2014.933411

[4] Zyriax B (2012) Welche nahrungsergänzungsmittel braucht die frau nach der menopause? J furGynakologische Endokrinol 6(3):5–11

[5] Dennehy C, Tsourounis C (2010) A review of select vitamins and minerals used by postmenopausal women. Maturitas 66(4):370–380 20580500 10.1016/j.maturitas.2010.06.003

[6] Bellows L, Moore R (2012) Water-soluble vitamins: b-complex and Vitamin c. colorado sate university extension food and nutrition series. Health 5(9):312

[7] Knovich MA, Storey JA, Coffman LG, Torti SV, Torti FM (2009) Ferritin for the clinician. Blood Reviews Mai 23(3):95–104 10.1016/j.blre.2008.08.001 PMC2717717 18835072

[8] Ma H, Lin H, Hu Y, Li X, He W, Jin X (2015) U. a. serum ferritin levels are associated with carotid atherosclerosis in Chinese postmenopausal women: the Shanghai Changfeng Study. Br J Nutr 114(7):1064–1071 26395322 10.1017/S0007114515001944

[9] Mackenzie EL, Iwasaki K, Tsuji Y (2008) Intracellular iron transport and storage: from molecular mechanisms to health implications. Antioxid Redox Signal Juni 10(6):997–1030 10.1089/ars.2007.1893 PMC2932529 18327971

[10] Rao A, Rao L (2007) Carotenoids and human health. Pharmacol Res März 55(3):207–216 10.1016/j.phrs.2007.01.012 17349800

[11] K.Debasish 2009 Colours of Health. African J Food Sci.3(5)

[12] Astorg P, Gradelet S, Bergès R, Suschetet M (1997) Dietary lycopene decreases the initiation of liver preneoplastic foci by diethylnitrosamine in the rat. Nutr Cancer Januar 29(1):60–68 10.1080/01635589709514603 9383786

[13] Paiva SAR, Russell RM (1999) β-carotene and other carotenoids as antioxidants. J Am Coll Nutr 18(5):426–433 10511324 10.1080/07315724.1999.10718880

[14] Johnson EJ (2002) The role of carotenoids in human health. Nutr Clin Care 5(2):56–65 12134711 10.1046/j.1523-5408.2002.00004.x

[15] Mayer-Miebach E, Behsnilian D (2010) Aspekte der herstellung haltbarer, lycopinreicher gemüse- und obstprodukte. J Verbr Lebensm Februar 5(1):51–58 10.1007/s00003-009-0305-8

[16] Chae M, Park K (2021) Association between dietary omega-3 fatty acid intake and depression in postmenopausal women. Nutr Res Pract 15(4):468 34349880 10.4162/nrp.2021.15.4.468 PMC8313386

[17] D. Vitamin (n.d.). DGE. [Internet]. Verfügbar unter:

[18] Office of dietary Supplements – Nutrient Recommendations and Databases. (n.d.). [Internet]. Verfügbar unter:

[19] Walallawita US, Wolber FM, Ziv-Gal A, Kruger MC, Heyes JA (2020) Potential role of lycopene in the prevention of postmenopausal bone loss: evidence from molecular to clinical studies. IJMS 21(19):7119 32992481 10.3390/ijms21197119 PMC7582596

[20] Ramakrishnan S, Sulochana KN, Lakshmi S, Ramakrishnan S, Sulochana KN, Lakshmi S (2006) U a biochemistry of homocysteine in health and diseases. Ind J Biochem Biophys 43(5):275–283 17133733

[21] Boushey CJ (1995) A quantitative assessment of plasma homocysteine as a risk factor for vascular disease: probable benefits of increasing folic acid intakes. JAMA 274(13):1049 7563456 10.1001/jama.1995.03530130055028

[22] Shenoy V, Mehendale V, Prabhu K, Shetty R, Rao P (2014) Correlation of serum homocysteine levels with the severity of coronary artery disease. Ind J Clin Biochem Juli 29(3):339–344 10.1007/s12291-013-0373-5 PMC4062675 24966483

[23] Ganguly P, Alam SF (2015) Role of homocysteine in the development of cardiovascular disease. Nutr J Dezember 14(1):6 10.1186/1475-2891-14-6 PMC4326479 25577237

[24] Hvas AM, Juul S, Bech P, Nexø E (2004) Vitamin b6 level is associated with symptoms of depression. Psychother Psychosom 73(6):340–343 15479988 10.1159/000080386

[25] Wu Y, Zhang L, Li S, Zhang D (2022) Associations of dietary Vitamin b1, Vitamin b2, Vitamin b6, and Vitamin b12 with the risk of depression: a systematic review and meta-analysis. Nutr Rev 80(3):351–366 33912967 10.1093/nutrit/nuab014

[26] Morris MS (2003) Homocysteine and Alzheimer’s disease. The Lancet Neurol Juli 2(7):425–428 10.1016/S1474-4422(03)00438-1 12849121

[27] Hu Q, Teng W, Li J, Hao F, Wang N (2016) Homocysteine and Alzheimer’s disease: evidence for a causal link from mendelian randomization. JAD 52(2):747–756 27031476 10.3233/JAD-150977

[28] Joosten E, Lesaffre E, Riezler R, Ghekiere V, Dereymaeker L, Pelemans W (1997) U a Is metabolic evidence for Vitamin b-12 and folate deficiency more frequent in elderly patients with alzheimer’s disease? J Gerontol A Biol Sci Med Sci 52A(2):M76–M79 10.1093/gerona/52A.2.M76 9060973

[29] Butola LK, Kute PK, Anjankar A, Dhok A, Gusain N, Vagga A (2020) Vitamin b12 - do you know everything? JEMDS 9(42):3139–3146 10.14260/jemds/2020/688

[30] Selhub J, Bagley LC, Miller J, Rosenberg IH (2000) B Vitamins, homocysteine, and neurocognitive function in the elderly. Am J Clin Nutr 71(2):614S-620S 10681269 10.1093/ajcn/71.2.614s

[31] Seshadri S, Beiser A, Selhub J, Jacques PF, Rosenberg IH, D’Agostino RB (2002) U a plasma homocysteine as a risk factor for dementia and Alzheimer’s Disease. N Engl J Med 346(7):476–483 11844848 10.1056/NEJMoa011613

[32] Muñoz-Torres M, Reyes-García R, Mezquita-Raya P, Fernández-García D, Alonso G, de Dios Luna J (2009) U a serum cathepsin K as a marker of bone metabolism in postmenopausal women treated with alendronate. Maturitas 64(3):188–192 19819089 10.1016/j.maturitas.2009.09.011

[33] Reynolds E (1976) The neurology of vitamin b12 deficiency metabolic mechanisms. The Lancet Oktober 308(7990):832–833 10.1016/S0140-6736(76)91213-7 61502

[34] Penninx BWJH (2000) Vitamin b12 deficiency and depression in physically disabled older women: epidemiologic evidence from the women’s health and aging study. Am J Psychiatry 157(5):715–721 10784463 10.1176/appi.ajp.157.5.715

[35] Hanna S, Lachover L, Rajarethinam RP (2009) Vitamin b12 deficiency and depression in the elderly: review and case report. Prim Care Companion J Clin Psychiatry 11(5):269–270 19956469 10.4088/PCC.08l00707 PMC2781043

[36] Zampatti S, Ricci F, Cusumano A, Marsella LT, Novelli G, Giardina E (2014) Review of nutrient actions on age-related macular degeneration. Nutr Res Februar 34(2):95–105 10.1016/j.nutres.2013.10.011 24461310

[37] Pérez-López FR, Chedraui P, Pilz S (2020) Vitamin D supplementation after the menopause. Therapeutic Adv Endocrinol Januar 11:1–13 10.1177/2042018820931291 PMC7278294 32551035

[38] Holick MF (2007) Vitamin D deficiency. N Engl J Med 357(3):266–281 17634462 10.1056/NEJMra070553

[39] Rizzoli R, Bischoff-Ferrari H, Dawson-Hughes B, Weaver C (2014) Nutrition and bone health in women after the menopause. Womens Health (Lond Engl) 10(6):599–608 25482487 10.2217/WHE.14.40

[40] Lips P, Vitamin D (2001) Deficiency and secondary hyperparathyroidism in the elderly: consequences for bone loss and fractures and therapeutic implications. Endocr Rev 22(4):477–501 11493580 10.1210/edrv.22.4.0437

[41] Lee JH, O’Keefe JH, Bell D, Hensrud DD, Holick MF (2008) Vitamin D deficiency. J Am Coll Cardiol 52(24):1949–1956 19055985 10.1016/j.jacc.2008.08.050

[42] Anderson JL, May HT, Horne BD, Bair TL, Hall NL, Carlquist JF (2010) U. a. relation of Vitamin d deficiency to cardiovascular risk factors, disease status, and incident events in a general healthcare population. Am J Cardiol 106(7):963–968 20854958 10.1016/j.amjcard.2010.05.027

[43] Mozos I, Marginean O (2015) Links between vitamin d deficiency and cardiovascular diseases. Biomed Res Int 2015:1–12 10.1155/2015/109275 PMC4427096 26000280

[44] Zacharski LR, Ornstein DL, Woloshin S, Schwartz LM (2000) Association of age, sex, and race with body iron stores in adults: analysis of NHANES III data. Am Heart J Juli 140(1):98–104 10.1067/mhj.2000.106646 10874269

[45] Milman N, Kirchhoff M (1992) Iron stores in 1359, 30–60 year-old danish women: evaluation by serum ferritin and hemoglobin. Ann Hematol Januar 64(1):22–27 10.1007/BF01811467 1739756

[46] Jian J, Pelle E, Huang X (2009) Iron and menopause: does increased iron affect the health of postmenopausal women? Antioxid Redox Signal Dezember 11(12):2939–2943 10.1089/ars.2009.2576 PMC2821138 19527179

[47] Zhang P, Wang S, Wang L, Shan BC, Zhang H, Yang F (2018) U. a. hepcidin is an endogenous protective factor for osteoporosis by reducing iron levels. J Mol Endocrinol 60(4):299–308 10.1530/JME-17-0301 29563156

[48] Zacharski LR, Shamayeva G, Chow BK (2011) Effect of controlled reduction of body iron stores on clinical outcomes in peripheral arterial disease. Am Heart J 162(5):949-957.e1 22093213 10.1016/j.ahj.2011.08.013

[49] JeromeL S (1981) Iron and the sex difference in heart disease risk. The Lancet Juni 317(8233):1293–1294 10.1016/S0140-6736(81)92463-6 6112609

[50] Huang X (2008) Does iron have a role in breast cancer? Lancet Oncol 9(8):803–807 18672216 10.1016/S1470-2045(08)70200-6 PMC2577284

[51] Li GF, Pan YZ, Sirois P, Li K, Xu YJ (2012) Iron homeostasis in osteoporosis and its clinical implications. Osteoporos Int Oktober 23(10):2403–2408 10.1007/s00198-012-1982-1 22525981

[52] Jian J, Yang Q, Dai J, Eckard J, Axelrod D, Smith J (2011) U. a. effects of iron deficiency and iron overload on angiogenesis and oxidative stress—a potential dual role for iron in breast cancer. Free Radical Biol Med 50(7):841–847 21193031 10.1016/j.freeradbiomed.2010.12.028 PMC3046244

[53] Yamasaki K, Hagiwara H (2009) Excess iron inhibits osteoblast metabolism. Toxicology Letters Dezember 191(2–3):211–215 10.1016/j.toxlet.2009.08.023 19735707

[54] Chen B, Li GF, Shen Y, Huang X, Xu YJ (2015) Reducing iron accumulation: a potential approach for the prevention and treatment of postmenopausal osteoporosis. Exp Thera Med Juli 10(1):7–11 10.3892/etm.2015.2484 PMC4486897 26170904

[55] Rivera S, Nemeth E, Gabayan V, Lopez MA, Farshidi D, Ganz T (2005) Synthetic hepcidin causes rapid dose-dependent hypoferremia and is concentrated in ferroportin-containing organs. Blood 106(6):2196–2199 15933050 10.1182/blood-2005-04-1766 PMC1895137

[56] Koren N, Simsa-Maziel S, Shahar R, Schwartz B, Monsonego-Ornan E (2014) Exposure to omega-3 fatty acids at early age accelerate bone growth and improve bone quality. J Nutr Biochem Juni 25(6):623–633 10.1016/j.jnutbio.2014.01.012 24746838

[57] Shen D, Zhang X, Li Z, Bai H, Chen L (2017) Effects of omega-3 fatty acids on bone turnover markers in postmenopausal women: systematic review and meta-analysis. Climacteric 20(6):522–527 29034731 10.1080/13697137.2017.1384952

[58] Kajarabille N, Díaz-Castro J, Hijano S, López-Frías M, López-Aliaga I, Ochoa JJ (2013) A new insight to bone turnover: role of -3 polyunsaturated fatty acids. Sci World J 2013:1–16 10.1155/2013/589641 PMC3834626 24302863

[59] Kruger MC, Horrobin DF (1997) Calcium metabolism, osteoporsis and essential fatty acids: a review. Prog Lipid Res 36(2–3):131–151 9624425 10.1016/S0163-7827(97)00007-6

[60] Griel AE, Kris-Etherton PM, Hilpert KF, Zhao G, West SG, Corwin RL (2007) An increase in dietary *n*-3 fatty acids decreases a marker of bone resorption in humans. Nutr J Dezember 6(1):2 10.1186/1475-2891-6-2 PMC1784104 17227589

[61] Fenton JI, Hord NG, Ghosh S, Gurzell EA (2013) Immunomodulation by dietary long chain omega-3 fatty acids and the potential for adverse health outcomes. Prostaglandins Leukot Essent Fatty Acids 89(6):379–390 24183073 10.1016/j.plefa.2013.09.011 PMC3912985

[62] Luchtman DW, Song C (2013) Cognitive enhancement by omega-3 fatty acids from child-hood to old age: findings from animal and clinical studies. Neuropharmacol Januar 64:550–565 10.1016/j.neuropharm.2012.07.019 22841917

[63] Agarwal S, Rao ΑV (2000) Carotenoids and chronic diseases. Drug Metab Drug Interact. https://doi.org/10.1515/DMDI.2000.17.1-4.189 10.1515/DMDI.2000.17.1-4.189 11201295

[64] Elliott R (2005) Mechanisms of genomic and non-genomic actions of carotenoids. (BBA) — Mole Basis Dis 1740(2):147–154 10.1016/j.bbadis.2004.12.009 15949681

[65] Fuhrman B, Elis A, Aviram M (1997) Hypocholesterolemic effect of lycopene and β-carotene is related to suppression of cholesterol synthesis and augmentation of ldl receptor activity in macrophages. Biochem Biophys Res Commun 233(3):658–662 9168909 10.1006/bbrc.1997.6520

[66] Rissanen T, Voutilainen S, Nyyssönen K, Salonen R, Salonen JT (2000) Low plasma lycopene concentration is associated with increased intima-media thickness of the carotid artery wall. ATVB Dezember 20(12):2677–2681 10.1161/01.ATV.20.12.2677 11116071

[67] Paran E (2001) Effect of tomato’s lycopene on blood pressure, serum lipoproteins, plasma homocysteine and oxidative sress markers in grade I hypertensive patients. Am J Hypertens 14(11):A141 10.1016/S0895-7061(01)01854-4

[68] Moriel P, Sevanian A, Ajzen S, Zanella MT, Plavnik FL, Rubbo H (2002) u. a. Nitric oxide, cholesterol oxides and endothelium-dependent vasodilation in plasma of patients with essential hypertension. Braz J Med Biol Res 35:1301–1309 12426629 10.1590/S0100-879X2002001100007

[69] Rao AV, Ray MR, Rao LG (2006) Lycopene. Adv Foof Nutr Res 51:99–164 10.1016/S1043-4526(06)51002-2 17011475

[70] Rao LG, Mackinnon ES, Josse RG, Murray TM, Strauss A, Rao AV (2007) Lycopene consumption decreases oxidative stress and bone resorption markers in postmenopausal women. Osteoporos Int Januar 18(1):109–115 10.1007/s00198-006-0205-z 16941193

[71] Palozza P, Simone RE, Catalano A, Mele MC (2011) Tomato lycopene and lung cancer prevention: from experimental to human studies. Cancers 3(2):2333–2357 24212813 10.3390/cancers3022333 PMC3757421

[72] Levi F, Pasche C, Lucchini F, Vecchia CL (2000) Dietary intake of selected micronutrients and breast-cancer risk. Int J Cancer 91(2):260–263 10.1002/1097-0215(200002)9999:9999<::AID-IJC1041>3.0.CO;2-# 11146455

[73] Hultén K, Van Kappel AL, Winkvist A, Kaaks R, Hallmans G, Lenner P (2001) U a carotenoids, alpha-tocopherols, and retinol in plasma and breast cancer risk in northern Sweden. Cancer Causes Control 12(6):529–537 11519761 10.1023/A:1011271222153

[74] Sato R, Helzlsouer KJ, Alberg AJ, Hoffman SC, Norkus EP, Comstock GW (2002) Prospective study of carotenoids, tocopherols, and retinoid concentrations and the risk of breast cancer. Am Assoc Cancer Res 11(5):451–457 12010859

[75] Peng SJ, Li J, Zhou Y, Tuo M, Qin XX, Yu Q (2017) U. a. in vitro effects and mechanisms of lycopene in MCF-7 human breast cancer cells. Genet Mol Res. https://doi.org/10.4238/gmr16029434 28407181 10.4238/gmr16029434

[76] Asbaghi S, Saedisomeolia A, Hosseini M, Honarvar NM, Khosravi A, Azargashb E (2015) Dietary intake and serum level of carotenoids in lung cancer patients: a case-control study. Nutr Cancer 67(6):893–898 26168284 10.1080/01635581.2015.1055365

[77] Park CK, Ishimi Y, Ohmura M, Yamaguchi M, Ikegami S (1997) Vitamin a and carotenoids stimulate differentiation of mouse osteoblastic cells. J J Nutr Sci Vitaminol 43(3):281–296 9268918 10.3177/jnsv.43.281

[78] Kim L, Rao AV, Rao LG (2003) Lycopene II—effect on osteoblasts: the carotenoid lycopene stimulates cell proliferation and alkaline phosphatase activity of SaOS-2 cells. J Med Food Juli 6(2):79–86 10.1089/109662003322233468 12935317

[79] Rao LG, Krishnadev N, Banasikowska K, Rao AV (2003) Lycopene I—effect on osteoclasts: lycopene inhibits basal and parathyroid hormone-stimulated osteoclast formation and mineral resorption mediated by reactive oxygen species in rat bone marrow cultures. J Med Food Juli 6(2):69–78 10.1089/109662003322233459 12935316

[80] Foy CJ (1999) Plasma chain-breaking antioxidants in alzheimer’s disease, vascular dementia and parkinson’s disease. QJM 92(1):39–45 10209671 10.1093/qjmed/92.1.39

[81] Schmidt R, Fazekas F, Hayn M, Schmidt H, Kapeller P, Roob G (1997) u a Risk factors for microangiopathy-related cerebral damage in the Austrian stroke prevention study. J Neurol Sci 152(1):15–21 9395122 10.1016/S0022-510X(97)00137-8

[82] Longnecker MP, Kamel F, Umbach DM, Munsat TL, Shefner JM, Lansdell LW (2000) U a dietary intake of calcium, magnesium and antioxidants in relation to risk of amyotrophic lateral sclerosis. Neuroepidemiology 19(4):210–216 10859501 10.1159/000026258

[83] Chen D, Huang C, Chen Z (2019) A review for the pharmacological effect of lycopene in central nervous system disorders. Biomed Pharmacother März 111:791–801 10.1016/j.biopha.2018.12.151 30616078

[84] Saini RK, Rengasamy KRR, Mahomoodally FM, Keum YS (2020) Protective effects of lycopene in cancer, cardiovascular, and neurodegenerative diseases: an update on epidemiological and mechanistic perspectives. Pharmacol Res Mai 155:104730 10.1016/j.phrs.2020.104730 32126272

[85] McCullough ML (2005) Dairy, calcium, and vitamin d intake and postmenopausal breast cancer risk in the cancer prevention study ii nutrition cohort. Cancer Epidemiol Biomark Prev 14(12):2898–2904 10.1158/1055-9965.EPI-05-0611 16365007

[86] Annweiler C, Rolland Y, Schott AM, Blain H, Vellas B, Herrmann FR (2012) U a higher Vitamin d dietary intake is associated with lower risk of Alzheimer’s disease: a 7-year follow-up. J Gerontol A Biol Sci Med Sci 67(11):1205–1211 22503994 10.1093/gerona/gls107

[87] Zacharski LR, Chow BK, Howes PS, Shamayeva G, Baron JA, Dalman RL (2007) U a reduction of iron stores and cardiovascular outcomes in patients with peripheral Arterial Disease a randomized controlled trial. JAMA 297(6):603 17299195 10.1001/jama.297.6.603

[88] Toniolo P (2001) Serum carotenoids and breast cancer. Am J Epidemiol 153(12):1142–1147 11415946 10.1093/aje/153.12.1142

[89] Ching S, Ingram D, Hahnel R, Beilby J, Rossi E (2002) Serum levels of micronutrients, antioxidants and total antioxidant status predict risk of breast cancer in a case control study. J Nutr 132(2):303–306 11823595 10.1093/jn/132.2.303

[90] Sesso HD, Buring JE, Zhang SM, Norkus EP, Gaziano JM (2005) Dietary and plasma lycopene and the risk of breast cancer. Am Assoc Cancer Res 14(5):1074–1081 10.1158/1055-9965.EPI-04-0683 15894655

